# A novel multi-biomarker combination predicting relapse from long-term remission after discontinuation of biological drugs in rheumatoid arthritis

**DOI:** 10.1038/s41598-021-00357-9

**Published:** 2021-10-21

**Authors:** Katsuya Nagatani, Eiji Sakashita, Hitoshi Endo, Seiji Minota

**Affiliations:** 1 Division of Rheumatology and Clinical Immunology, Department of Internal Medicine, Jichi Medical University School of Medicine, Tochigi, Japan; 2 Department of Biochemistry, Jichi Medical University School of Medicine, Tochigi, Japan

**Keywords:** Rheumatoid arthritis, Predictive markers

## Abstract

Biological disease modifying anti-rheumatic drugs (bDMARDs) show dramatic treatment efficacy in rheumatoid arthritis (RA). Long-term use of bDMARDs, however, has disadvantages such as high costs and infection risk. Therefore, a methodology is needed to predict any future RA relapse. Herein, we report a novel multi-biomarker combination which predicts relapse after bDMARDs-withdrawal in patients in remission. Forty patients with RA in remission for more than 12 months were enrolled. bDMARDs were withdrawn and they were followed monthly for the next 24 months. Fourteen patients (35%) of 40 in the cohort remained in remission at 24 months, whereas 26 (65%) relapsed at various time-points. Serum samples obtained longitudinally from patients in remission were assessed for the relapse-prediction biomarkers and index from 73 cytokines by the exploratory multivariate ROC analysis. The relapse-prediction index calculated from the 5 cytokines, IL-34, CCL1, IL-1β, IL-2 and IL-19, strongly discriminated between patients who relapsed and those who stayed in remission. These findings could contribute to clinical decision-making as to the timing of when to discontinue bDMARDs in RA treatment.

## Introduction

Since the advent of etanercept in 1998 in the United States and infliximab in 2003 in Japan as the first biological disease modifying anti-rheumatic drugs (bDMARD), treatment of patients with rheumatoid arthritis (RA) has been dramatically changed with unprecedented efficacy. RA prognosis is much better now and many patients with RA achieve quality of life equal to that before disease development. Indeed, if RA patients are treated at an early stage, some patients report feeling cured^1^.

However, bDMARDs are much more expensive than conventional synthetic DMARDs, and economic status may cause patients to forgo the best modality of RA treatment even in Japan where all residents are covered by national health insurance^2^.

Aside from cost, the risk of infection is another important consideration. Early on, when tumor necrosis factor inhibitors were first used, infections such as tuberculosis, atypical mycobacteriosis and *Pneumocystis jirovecii* pneumonia were a serious risk. Although the risk of infection is now less, the risks remain; patients as well as their physicians need to be alert to the danger of infection which can be serious and potentially fatal^3^.

Because of these concerns, patients on bDMARDs and in remission may choose to discontinue them. However, disease exacerbation or relapse occurs in a sizable number of patients after withdrawal of bDMARDs. Relapse is unpredictable and may be insidious. Resumption of bDMARDs is usually needed to control disease flare-ups. Further, joint pain and destruction can progress considerably as the resumption of bDMARDs may take longer to show effect. Therefore, one of the unmet needs in RA treatment to be addressed urgently is what the likelihood of relapse will be after the withdrawal of bDMARDs. To date, there are few publications which have addressed this problem^4^. Several clinical parameters were developed to evaluate RA disease-activity for clinical practice such as the Disease Activity Score (DAS), the modified DAS using 28 joints (DAS28)^5–7^, the Clinical Disease Activity Index (CDAI)^8^, the Simplified Disease Activity Index (SDAI)^9^, and the Boolean-based index as the most stringent^5^. These parameters were based upon tender and swollen joint counts, patients’ self-assessment and inflammatory biomarkers in blood. However, the major obstacles of these parameters are intra-/inter-observer variability and inconsistent reporting by patients. To overcome these issues, another parameter was developed which utilizes 12 serum biomarkers: the multiple biomarker disease activity (MBDA) score. The MBDA score standardises RA disease activity on the scale of 1–100^10^. The MBDA score correlates with exacerbation and predicts exacerbation-probability after withdrawal of tumor necrosis factor inhibitor treatment in RA patients with stable low-grade disease activity^11^. However, most of the 12 serum biomarkers in the MBDA score, including CRP, SAA, MMP-1, MMP-3, IL-6, leptin, etc., reflect the inflammation level of the patients at the time of the measurement^10^. Therefore, it may not be appropriate to use for the relapse-prediction for the patients in long-term remission without any biomarkers indicative of inflammation.

In the present study, we pursued biomarkers to be used to predict RA-relapse after withdrawal of bDMARDs in patients in long-term remission (longer than 1 year) by following prospectively and collecting serum samples longitudinally. A biomarker combination of five cytokines was identified through comprehensive analysis of multiple biomarkers and exploratory ROC analysis. This combination could potentially discriminate patients in remission who relapse within 2 years of bDMARDs-withdrawal from those who maintain remission. The biomarkers, which were quite different from those in the MBDA score, and the calculated index, would be useful for clinical decision-making as to whether to discontinue bDMARDs while in remission.

## Results

### Patient demographics

Forty patients in remission (DAS28-CRP < 2.3) for at least 1 year by bDMARDs-treatment were enrolled and bDMARDs were discontinued at the time of study initiation; 14 patients remained in remission for 2 years and 26 relapsed at some point in time. DAS28-CRP is a variation of DAS28 in which serum levels of C-reactive protein replace those of sedimentation rates. The patient demographics are summarized in Table 1. The relapsed group had a higher percentage of multiple bDMARDs-user and lower levels of serum CRP. However, these characteristics did not reach statistical significance. The DAS28-CRP score was statistically higher in the non-relapsed group. Although the initial condition for patient recruitment was DAS28-CRP < 2.3 for at least 1 year, it was found retrospectively all the participants fulfilled the Boolean remission criteria at the time of study initiation.

**Table 1:** Patients demographics.

Characteristics	Total population (n = 40)		
	Sustained remission (n = 14)	Relapse (n = 26)	*p* values
Age, years	60 (39–63)	59 (45–66)	0.98^a^
Female gender, n (%)	10 (71.4)	22 (84.6)	0.28^b^
Disease duration, years	5.0 (3.0–7.5)	6.5 (5.0–11.8)	0.15^a^
Radiographic stage III or IV, n (%)^c^	2 (14.3)	8 (30.8)	0.23^b^
Number of bDMARDs used before study initiation, n (%)			0.07^b^
1	13 (92.9)	17 (65.4)	
≥ 2	1 (7.1)	9 (34.6)	
Remission duration, months	41.5 (25.8–52.0)	47.5 (26.3–61.3)	0.31^a^
Methotrexate dose, mg/week	6.0 (4.0–8.0)	5.0 (0.5–8.0)	0.79^a^
Prednisolone dose, mg/day^d^	0.0 (0.0–0.0)	0.0 (0.0–0.0)	0.15^a^
Seropositive (RF or ACPA), n (%)	13 (92.9)	23 (88.5)	0.56^b^
CRP (mg/dL)	0.05 (0.03–0.11)	0.03 (0.02–0.06)	0.08^a^
SAA (μg/mL)	0.0 (0.0–2.1)	0.0 (0.0–0.0)	0.57^a^
DAS28-CRP before treatment with bDMARDs	4.00 (3.50–4.38)	3.93 (2.96–5.31)	0.72^a^
DAS28-CRP at study initiation	1.29 (1.11–1.40)	1.09 (1.04–1.19)	0.02^a^
Boolean remission, n (%)	14 (100)	26 (100)	0.59^b^
TNF inhibitors, n (%)			0.30^b^
Infliximab	4 (28.6)	9 (34.6)	
Etanercept	7 (50.0)	10 (38.5)	
Adalimumab	2 (14.3)	2 (7.7)	
IL-6 inhibitor, n (%)			
Tocilizumab	1 (7.1)	5 (19.2)	

Values are presented as median (inter-quartile range; IQR) unless otherwise specified. ^a^Mann–Whitney’s *U* test. ^b^Fisher’s exact probability test. ^c^Steinbrocker stage definition^35^. ^d^None of the patients in the sustained remission group was taking prednisolone, and 4 in the relapse group were taking prednisolone at 1 mg in one, 2 mg in two and 3 mg in one at the study initiation. The amount of prednisolone was unchanged throughout the study period. Statistical analysis was performed using EZR^31^.

bDMARDs used to induce and sustain remission for at least 1 year are also shown in Table 1. Thirteen patients were treated with infliximab, 17 with etanercept, 4 with adalimumab, and 6 with tocilizumab. Although the relapse rate seemed to be higher in patients treated with tocilizumab compared to those who received anti-TNF-α agents, there was no difference statistically.

**Figure 1:**
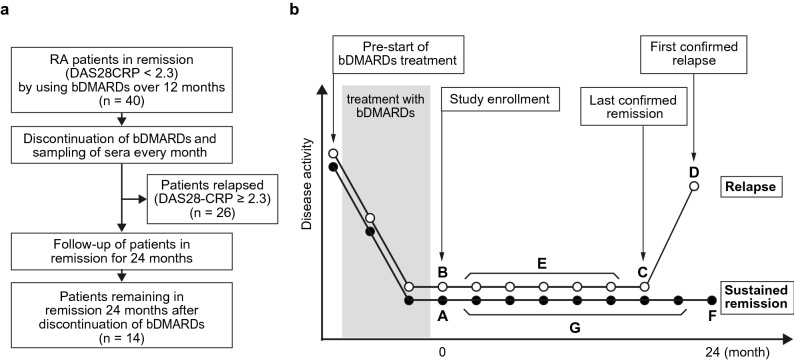
Study design. (**a**) Flow chart of patient enrollment. RA patients in remission judged by DAS28-CRP for at least 12 months by using bDMARDs were included. After discontinuation of bDMARDs, those patients were followed, and serum samples were collected monthly. Follow-up period spanned up to 24 months if the patients stayed in remission. (**b**) Schema of the timing of clinic-visits and serum sampling. Points A and B are the time of study initiation for the non-relapsed (sustained remission) and relapsed groups, respectively. Points C is the time of the last confirmed remission and D is the time of the first confirmed relapse and the study-end in the relapsed group. E points are the time of monthly clinic-visits in the relapsed group while in remission. Point F is the time of the study-end for the non-relapsed group. G points are the time of monthly clinic-visits in the non-relapsed group. The images were created using Adobe Illustrator (ver. 25.4.1, https://www.adobe.com/).

**Figure 2:**
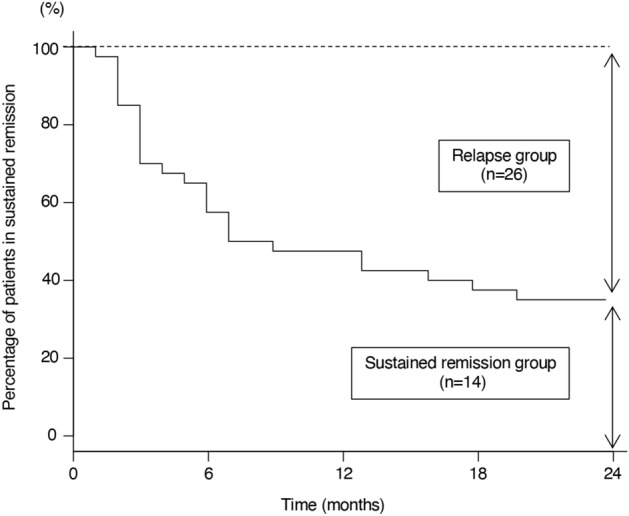
Kaplan–Meier curve showing percentage of patients on remission during 24 months of study span. The number of patients in remission decreased quickly during the first 6 months and the reduction rate became very slow thereafter. The image was created using EZR (ver. 1.52, https://www.jichi.ac.jp/saitama-sct/SaitamaHP.files/statmedEN.html).

Overall, patients in the relapsed and sustained remission (non-relapsed) groups had moderate disease activity with DAS28-CRP score of 4.00 (3.50–4.38) and 3.93 (2.96–5.31), respectively, before initiation of bDMARDs. The DAS28-CRP score was very low at 1.29 (1.11–1.40) and 1.09 (1.04–1.19) in the relapsed and non-relapsed groups, respectively, at the time of study initiation. Over a period of 24 months after bDMARDs-withdrawal, patients whose DAS28-CRP score became ≥ 2.3 were classified as relapsed, and the study was terminated in those patients (Fig. 1a) with treatment fortification to treat the relapse. The rate of remission declined steeply to ~ 50% at 6 months, slowly to ~ 40% at 12 months, and finally to ~ 35% at 24 months according to the Kaplan–Meier estimate (Fig. 2). In short, the relapse rate nearly plateaued after 12 months.

### Difference of cytokine profiles between relapsed and non-relapsed groups

**Figure 3:**
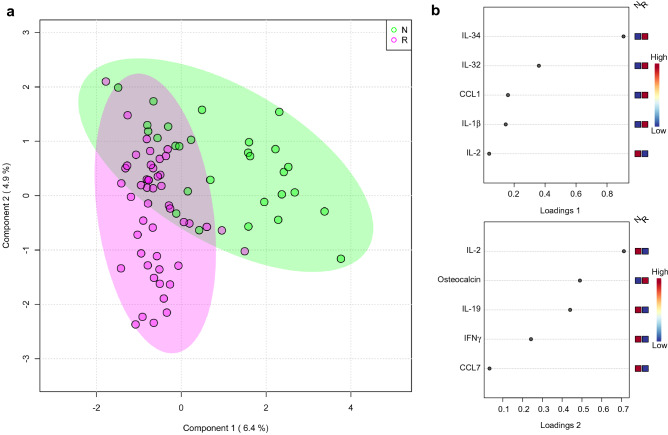
sPLS-DA plot showing model discrimination between relapsed and non-relapsed groups with a partial overlap. (**a**) sPLS-DA 2D plot of the relapsed (R) and non-relapsed (N) groups. (**b**) Loading plot indicating the most discriminating biomarkers of component 1 (upper panel) and component 2 (lower panel) in descending order of contribution. The serum concentration of the analyte was measured in each patient at time points A and F in the sustained remission group and at time points B and C in the relapse group (Fig. 1). The images were created using MetaboAnalyst (ver. 4.0, https://www.metaboanalyst.ca).

Quantification of 73 cytokines (Table S1) in sera from patients relapsed (R) (n = 48: time points B and C in Fig. 1b and Table S2) and those with sustained remission (non-relapsed; N) (n = 28: time points A and F in Fig. 1b and Table S2) were performed with multi-plex cytokine/chemokine arrays sold by Bio-Rad Laboratories. Data normalization was carried out to reduce any systematic bias through the given data points and to provide a consistent biological comparison. The patients who relapsed and those in sustained remission could be distinguished using sparse partial least squares discriminant analysis (sPLS-DA) plot (Fig. 3a) using MetaboAnalyst^12^ according to their cytokine profiles, showing a group separation with a partial overlap on the two component axes. These results suggest that the cytokine profiles in serum of the relapsed group were very different from those in the sustained remission group. Next, variable importance in projection (VIP) scores for multiplex cytokine profiles in sPLS-DA was calculated (Fig. 3b). We identified the top 5 cytokines on component 1 and 2, respectively. Half of the selected 10 cytokines, IL-34, IL-32, CCL1, IL-1β, and osteocalcin, were found to be significantly upregulated in samples from the relapsed group compared to those from the sustained remission group, while the remaining four, IL-2, IL-19, IFNγ, and CCL7, were notably downregulated.

**Figure 4:**
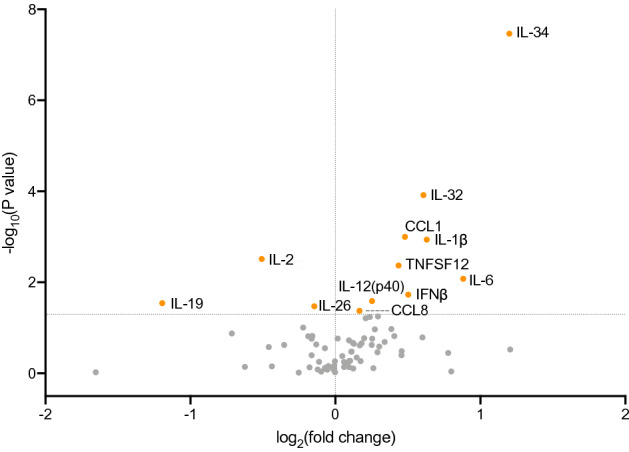
Volcano plot displaying biomarkers with a significant *p-*value (y axis) and fold change (relapse/non-relapse, x axis) between relapsed and non-relapsed groups at study initiation. The horizontal dotted line indicates where *p* = 0.05. Biomarkers with *p* value of < 0.05 are shown by orange dots. The serum concentration of the analyte was measured in each patient at time points A and F in the sustained remission group and at time points B and C in the relapse group (Fig. 1). The image was created using GraphPad Prism 8 (http://www.graphpad.com).

A volcano plot was drawn to visualize the cytokines with a significant difference in serum samples between the relapsed and sustained remission groups. We identified 12 cytokines in a feature selection based on *t*-test probabilities (*p* value < 0.05 in Fig. 4 and Table 2). A fold change of > 2.0 was set arbitrarily to be significant. We found two cytokines with a significant difference (*p* value < 0.05, fold change > 2.0): IL-34 as up-regulated and IL-19 as down-regulated in the relapsed group.

**Table 2:** Fold change of significant biomarkers.

Analyte	log_2_(fold change)	*p* value	Adjusted *p* value
IL-34*	1.200	3.42E−08	3.18E−06
IL-32*	0.608	1.21E−04	5.62E−03
CCL1*	0.480	1.00E−03	2.68E−02
IL-1β*	0.630	1.15E−03	2.68E−02
IL-2*	− 0.508	3.06E−03	5.69E−02
TNFSF12	0.436	4.25E−03	6.59E−02
IL-6	0.882	8.38E−03	1.11E−01
IFNβ	0.503	1.87E−02	2.17E−01
IL-12 (p40)	0.252	2.58E−02	2.66E−01
IL-19*	− 1.194	2.86E−02	2.66E−01
IL-26	− 0.146	3.37E−02	2.85E−01
CCL8	0.167	4.24E−02	3.29E−01

A positive fold change indicates a higher concentration in the relapse group.

*High contribution features by the sPLS-DA. The serum concentration of the analyte was measured in each patient at time points A and F in the sustained remission group and at time points B and C in the relapse group (Fig. 1).

### Protein–protein interaction (PPI) analysis to identify functional networks

**Figure 5:**
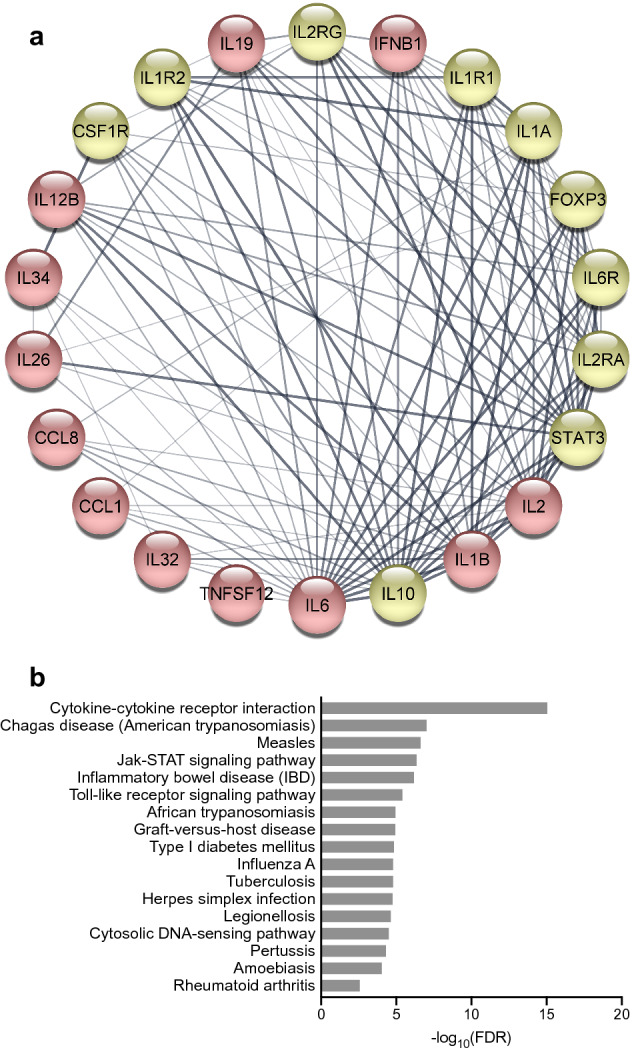
Relapse-associated biomolecular network. (**a**) Protein–protein interaction (PPI) networks of relapse-associated 12 target proteins shown in red were analyzed by STRING database with a confidence score of 0.4, and ten more related targets shown in yellow were identified. Line thickness indicates the strength of data support. (**b**) Top 16 pathways and rheumatoid arthritis pathways (ranked 28th out of 41 pathways) from KEGG pathway analysis of 12 relapse-associated proteins. X-axis (in bar graphs) indicates the significance (− log_10_[FDR]) of the pathway association. The PPI image was created using Cytoscape (ver. 3.8.2, https://cytoscape.org). The pathway analysis image was created using GraphPad Prism 8 (http://www.graphpad.com).

To better understand the interplay among the identified 12 cytokines in the *t*-test shown above (Fig. 4), we obtained potential protein–protein interaction (PPI) network using the STRING database (Fig. 5a). The PPI network consists of 22 nodes including 10 additional relevant proteins, and 120 edges. Most protein interactions are related to IL-6, IL-10, IL-1β, IL-2, and STAT3, while information is limited regarding the interaction profile of TNSF12, IL-32, CCL1, and CCL8. The interaction between IL-34 and CSF1R provided the highest combined score, which is STRING’s confidence score calculated from all the contributing evidence. The interaction of IL-19 with STAT3 or IL-26 also provided a high or moderate combined score, respectively. To get further insight into the signaling pathway that mediates the relapse, we further performed pathway analysis using Kyoto Encyclopedia of Genes and Genomes (KEGG) database. We found that these genes were involved in infectious disease responses such as Chagas disease, measles, influenza A, and tuberculosis as well as immune-mediated disorders such as inflammatory bowel disease, graft-versus-host disease, and type I diabetes (Fig. 5b). A similar immune pathway might be at work in rheumatoid arthritis.

### Univariate receiver operating characteristic curve (ROC) analysis

**Figure 6:**
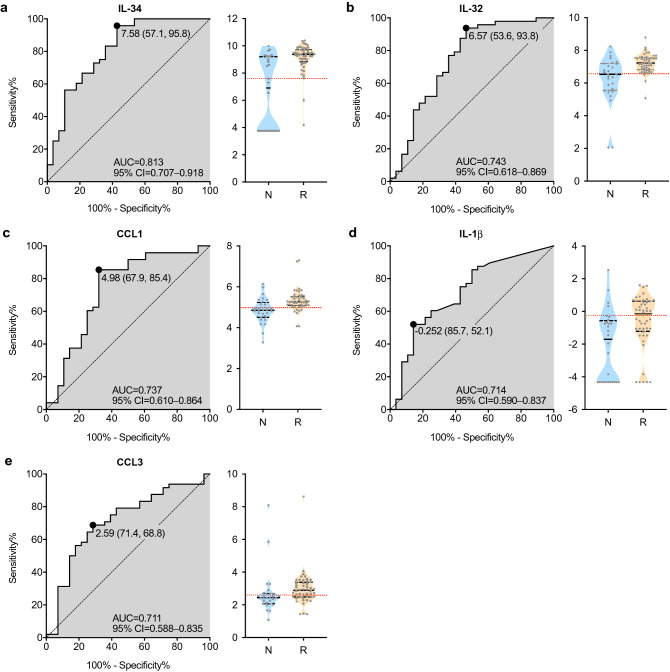
ROC curves and violin plots of the selected biomarkers. Serum levels of IL-34 (**a**), IL-32 (**b**), CCL1 (**c**), IL-1β (**d**), and CCL3 (**e**) are higher in the relapsed group than in the non-relapsed group. ROC curves (left) and violin plots (right) show the distribution of the log_2_ transformed levels of biomarkers in relapse (R, orange) and non-relapse (N, light blue) samples. The area under the curve (AUC) for each of the ROC curves is annotated with the 95% confidence interval (CI) by Wilson/Brown method. The best threshold measured using “farthest to diagonal line” method with sensitivity and specificity was also shown as a dot on ROC curve and as a red dotted line in the violin plot. The serum concentration of the analyte was measured in each patient at time points A and F in the sustained remission group and at time points B and C in the relapse group (Fig. 1). The images were created using GraphPad Prism 8 (http://www.graphpad.com).

Univariate ROC curve analysis was performed to evaluate the diagnostic power of best performing cytokines for prediction of future relapse in patients in remission. The cytokine concentration data, which are log-transformed but without autoscaling, were used for the analysis. When the value of area under the curve (AUC) was > 0.7, five cytokines, IL-34, IL-32, CCL1, IL-1β, and CCL3 were identified as potential biomarkers. The AUC was 0.813 for IL-34, 0.743 for IL-32, 0.737 for CCL1, 0.714 for IL-1β, and 0.711 for CCL3 (Fig. 6 and Table 3). The top four of the AUC-selected 5 cytokines were common to those selected in sPLS-DA (Fig. 3) and *p*-value (Fig. 4) methods. In both, autoscaled concentration data were used for normalization. Univariate ROC analysis could not identify cytokines with high AUC, specificity, and sensitivity (> 0.8, > 80% and > 80%, respectively), suggesting that a selected individual cytokine might not be high enough for discrimination on its own.

**Table 3:** Comparison by ROC analysis of significant biomarkers.

Analyte	AUC	95% CI	Sensitivity (%)	Specificity (%)	Cut off (pg/mL)
IL-34	0.813	0.707–0.918	95.8	57.1	192
IL-32	0.743	0.618–0.869	93.8	53.6	95.0
CCL1	0.737	0.610–0.864	85.4	67.9	31.5
IL-1β	0.714	0.590–0.837	52.1	85.7	0.840
CCL3	0.711	0.588–0.835	68.8	71.4	6.01

### Exploratory multivariate ROC analysis

**Figure 7:**
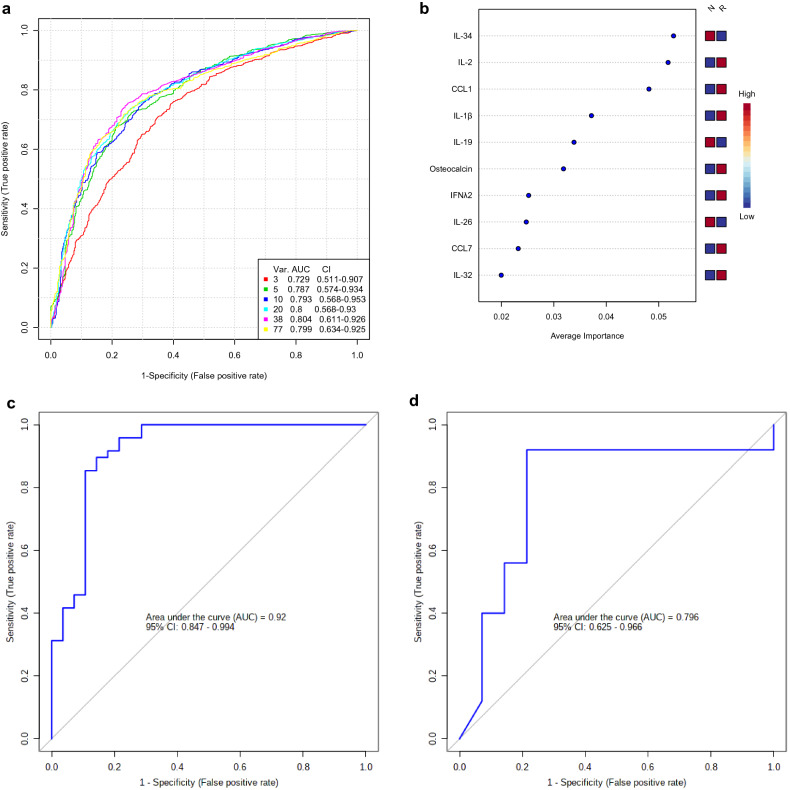
Selection of the significant five biomarkers. (**a**) Comparison of six ROC curve models created using linear support vector machine (SVM) algorithm with different numbers of features (3, 5, 10, 20, 38 and 77). The legend shows the AUCs and confidence intervals (CI) of the six models. (**b**) Top 10 significant biomarkers ranked based on mean importance measure in descending order of importance. (**c**) ROC curve plot from five biomarker combination created using logistic regression algorithm with tenfold cross-validation. The ROC curve was generated using a combination of IL-34, IL-2, CCL1, IL-1β, and IL-19, which are selected as top five significant biomarkers in (**b**). The serum concentration of the analyte was measured in each patient at time points A and F in the sustained remission group and at time points B and C in the relapse group (Fig. 1). (**d**) ROC curve evaluation of the five-biomarker model using serum from the relapsed and non-relapsed patients at bDMARDs-withdrawal. The ROC curve was generated as described in (**c**). The serum concentration of the analyte was measured in each patient at time point A in the sustained remission group and at time point B in the relapse group (Fig. 1). The images were created using MetaboAnalyst (ver. 4.0, https://www.metaboanalyst.ca).

To improve the discriminatory power, a multivariate ROC curve analysis using linear support vector machine algorithm was performed to identify a minimum number of features model and the feature selection. The combination of 5 features was validated as a good model for the prediction of high power. A 5 feature model had as similar an AUC value (0.787) as that of more than 10 features (Fig. 7a). From the VIP plot, IL-34, IL-2, CCL1, IL-1β and IL-19 were selected as top 5 features (Fig. 7b). Notably, top four cytokines of IL-34, IL-2, CCL1, and IL-1β were common to those in sPLS-DA and *p*-value methods.

To evaluate a combination of the 5 feature model, we performed ROC curve plot analysis using logistic regression algorithm with tenfold cross-validation. The top 5 feature combination revealed better performance of this model (AUC = 0.92, Fig. 7c) for relapse prediction than those of single feature model in Fig. 6. The predicted probability of relapse using five features (relapse prediction index score; RPI score) can be calculated as follows: RPI score = logit(*P*) = − 21.228 + 0.791(*IL-34*) + 4.252 (*CCL1*) − 2.419 (*IL-2*) + 0.465 (*IL-1β*) − 0.18 (*IL-19*), where *IL-34, CCL1*, *IL-2, IL-1β* and *IL-19* are log-transformed serum concentration. The RPI score formula was established via multivariate logistic regression model, logit(P) expression, where P is the estimated probability of relapse occurrence. The details of this logit regression model are given in Table S3. The cut-off value is 0.630. If the result of the RPI score is ≥ 0.630, the patient with this sample is predicted to relapse in the future; otherwise, the patient is classified as a non-relapser.

To examine whether the 5 predictor set model drawn from two time points (“A” and “F” for the non-relapsed and “B” and “C” for the relapsed group in Fig. 1b) can be applied to a data set from a single time point, we performed ROC curve plot analysis using the data set consisting of “A (n = 14)” and “B (n = 25).” As expected, the five-predictor set showed high performance in the prediction of future relapse using sera from patients only at the time of bDMARDs-withdrawal (AUC = 0.796, Fig. 7d), indicating the stability of the prediction from the five data set if remission continues. Furthermore, when this data set was applied to the calculation of the RPI, the value of RPI was 0.880 (95% CI 0.688–0.975) for sensitivity, 0.857 (95% CI 0.572–0.982) for specificity, and 6.16 (95% CI 1.69–22.4) for positive likelihood ratio. If the validation by the external cohorts endorses our results, RPI might be helpful for clinical decision-making.

## Discussion

Since it is very difficult to predict prospectively exacerbation or relapse in RA at the time of bDMARDs-withdrawal, hesitation is the rule in the decision to withdraw or continue bDMARDs in patients in long-term remission. To address this hesitancy, patients in clinical remission for at least 1 year on bDMARDs were recruited, their bDMARDs discontinued (40 patients), and were then followed for 2 years. Twenty-six patients out of 40 eventually relapsed whereas 14 patients stayed in remission during the 2-year follow-up. By comparing these two groups, we found a biomarker combination consisting of five cytokines which could predict, at the time of bDMARDs-withdrawal, the future relapse of RA. These were IL-34, IL-2, CCL1, IL-1β and IL-19. They emerged from the process of comprehensive, longitudinal analyses of serum cytokines employing exploratory ROC analysis. The five factors did not include biomarkers of RA inflammation such as CRP, SAA or MMP-3. The levels of serum CRP and SAA were measured in all the patients as clinical parameters at every clinic visit; these were included in the analysis. Although statistically insignificant, the number of the patients receiving tocilizumab in the relapse group was higher than that in the sustained remission group. Tocilizumab suppresses serum levels of CRP through its biological action. However, CRP levels in the patients who had been on tocilizumab stayed in the normal range after tocilizumab-withdrawal until joint pain/swelling appeared and the relapse was confirmed. Therefore, contribution of CRP levels to the DAS28-CRP relapse seemed to be minimal in the patients who had been treated with tocilizumab.

The five factors would not be disease activity markers, but relapse prediction markers unrelated to inflammatory status. This is quite different from earlier reports such as the MBDA score^4,10,13^. Patients in our cohort were in long-term remission making acute phase reactants or cartilage destructive factor less likely to feature. To date, DAS-28, SDAI and CDAI have been used to evaluate levels of RA disease activity at a designated time point. In addition, another score, the multi-biomarker disease activity (MBDA) score was developed to evaluate more objectively levels of RA disease activity. By applying the MBDA score, it was also useful to predict future exacerbation of RA after withdrawal or reduction of treatments^14^. It is quite reasonable to assume that patients with higher RA disease activity have a higher chance of exacerbation after withdrawal of bDMARDs or a shorter period until an exacerbation occurs. In this regard, the MBDA score is useful for an exacerbation prediction marker. However, for patients in long-term remission, the MBDA score might be less effective as a relapse predictor because factors related to disease activity and inflammation are quite low or normal in this clinical setting.

There are several reports on the relapse rates after bDMARDs-withdrawal. Follow-up periods were relatively short, mostly 6 months and 12 months at the longest^11,15^, with the exception of one recent report^4^. The reported data at 6 and 12 months are very similar to ours: ~ 50% relapsed at 6 months and ~ 60% at 12 months. However, to our surprise, the relapse rate almost plateaued after 12 months: ~ 60% at 12 months and ~ 65% at 24 months. The recently published study showed similar results of long-term follow-up to ours^4^. The fact that more than 30% of the patients did not relapse for a long time after bDMARDs-withdrawal implies that a predictable discriminator is very important clinically. These patients could be off bDMARD, reducing the cost-payment and infection risk.

In the present study, we identified the multi-biomarker combination which could predict future relapse by comprehensive exploratory multivariate analysis, post hoc, of the data from the patients in remission. After withdrawal of bDMARDs, all the patients were followed by an expert rheumatologist (KN) and their serum samples were stored longitudinally every month. To level disease activity during remission, we chose two serum samples from each patient: the point of bDMARD-withdrawal and the point immediately prior to relapse or at the last visit for the non-relapser. The measurements from these two points in each patient were used initially to select the biomarker combination for relapse prediction. However, the same biomarker combination was also found to be useful from the measurements only at the point of bDMARDs-withdrawal (Fig. 7d), suggesting that if patients had been in prolonged remission and biochemically stable, the level of calculated prediction was also stable.

This might have profound clinical implication. In clinical settings, the accurate prediction of future relapse is most helpful when it is feasible at the time of treatment change typified by bDMARDs-withdrawal in RA. By applying the biomarker combination and the index drawn from them (RPI score ≥ 0.63 for relapse), patients and their rheumatologists would be able to discuss and make shared and informed decisions early in the course of treatment with more confidence. It is also noteworthy that most of the five features selected through VIP plot were also selected by sPLS-DA and *p*-value methods, consolidating the data probability.

IL-34, CCL1, and IL-1β might promote or aggravate RA disease processes. On the other hand, IL-2 and IL-19 correlated inversely with the relapse. It is intriguing that IL-2 is independently selected in the same line of study published recently^4^. IL-19 belongs to the IL-10 family and is immunosuppressive. IL-19 down-regulates inflammation and might have inhibitory effects on the RA disease process^16,17^. IL-2 has a distinctive characteristic. IL-2 stimulates and proliferates T cells through its receptor binding and signaling. However, IL-2 knockout mice are sometimes autoimmune and produce autoantibodies of different kinds depending on the mouse background. They sometimes show autoimmune disorders which are occasionally lethal^18^. IL-2 is indispensable for the integrity of regulatory T cells^19^. It is very interesting and intriguing that both aggravating and inhibitory factors were selected as the relapse prediction biomarkers in this study, indicating several processes being at work in the exacerbation/relapse of RA^20^.

As shown in Fig. 5a, the selected five biomarkers interconnect each other, suggesting that they are all relevant biomarkers. IL-34 showed the strongest correlation with RA relapse after bDMARDs-withdrawal and in this regard we consider IL-34 as pivotal. IL-34 is a ligand to colony-stimulating factor-1 receptor (a.k.a. M-CSF receptor). IL-34 is detected at high levels in patients with active RA and in experimental models of inflammatory arthritis^21^. Crucial roles of IL-34 are shown in the proliferation and differentiation of mononuclear phagocyte lineage cells^22^, osteoclastogenesis^23^, inflammation^24,25^, angiogenesis^26^, and cell adhesion and migration^27^. All of these are very important in the pathology and pathophysiology of RA. Additionally, blockade of IL-34 by a specific monoclonal antibody reduces the severity of inflammatory arthritis in mice, implying a novel therapeutic potential^28^. IL-1β and TNF-α enhance IL-34 expression in synovial fibroblasts of RA patients and IL-34 is proposed to be working downstream of these cytokines^29^. Our finding might be in accordance with these previous findings. In the present study, patients with low levels of serum IL-34 while in remission were found to have a better chance to maintain remission after bDMARDs-withdrawal^30^. That is to say, serum IL-34 could be a small pilot fire which, in itself, is not enough to induce elevation of an inflammatory response and biomarkers such as CRP, SAA and MMP-3, in remission. However, it is enough to rekindle the inflammatory fire of RA once the effect of bDMARDs is removed. Imbalance between pro-inflammatory cytokines such as IL-34 and anti-inflammatory cytokines such as IL-19 would drive RA disease activity towards exacerbation or relapse. AUC from univariate analysis of IL-34 might not be so high. Therefore, it is appropriate and reasonable to use this combination of several biomarkers as we have shown to calculate the probability of relapse. Application of the RPI score to RA patients is as easy as application of the DAS28-CRP in the routine clinical setting.

Though the number of patients in our cohort was small, we were still able to select the biomarker combination composed of five cytokines and the index drawn from them which would predict future relapse. Patients in our cohort had been in remission for a long time. All the patients fulfilled the Boolean criteria for remission. This made them relatively uniform and suitable for the present study. This might be the reason for an excellent AUC (= 0.92) when the calculation was performed using two measurement points in each patient: one at the time of bDMARDs-withdrawal and another at the last confirmed remission in the relapse group or final point in the sustained remission group. However, cytokine measurement only at bDMARDs-withdrawal point in each patient is pivotal in our research, and this made AUC lower to 0.796. Still, we think it is adequate in the clinical standpoint (Fig. 7c,d).

It is interesting that patients in remission can be dichotomized into those who relapse and those who maintain remission after treatment reduction; our study might have shown one of the reasons for this. However, the relapse-predictive biomarker combination and the logistic regression model must be validated and consolidated using a different cohort and this study is now under way.

## Methods

### Study design, patients and sera

Forty patients with rheumatoid arthritis (RA) who met the 2010 American College of Rheumatology/European League Against Rheumatism (ACR/EULAR) criteria for RA were prospectively enrolled. They were treated with bDMARDs (either tumor necrosis factor inhibitor or IL-6 receptor blocker) and had maintained clinical remission in conformity with DAS28-CRP < 2.3 for more than 12 months. After bDMARDs were withdrawn from the treatment regimen, the patient’s remaining medication protocol stayed unchanged up to the study end. None of the patients in the sustained remission group was taking prednisolone, and 4 in the relapse group were taking prednisolone at 1 mg in one, 2 mg in two and 3 mg in one at the study initiation (Table 1). The amount of prednisolone was unchanged and intra-articular glucocorticoids were not allowed throughout the study period. The patients were evaluated for disease activity and their serum samples were collected approximately every 4 weeks. They were instructed to visit the clinic anytime when they felt any joint problems. If the disease activity score of a patient became higher than that of low disease activity score (DAS28-CRP ≥ 2.3), the patient was considered to have relapsed, and the study came to an end for that patient. If patients stayed in remission, they were followed for 24 months.

This study was conducted in compliance with the Helsinki Declaration. The Institutional Review Board of Jichi Medical University approved this study, and patients gave their written informed consent before being enrolled in the study. This study was registered in the University Hospital Medical Information Network Clinical Trials Registry (UMIN000044434)*.*

### Statistical analysis

Statistical analyses for demographics of the patients (Table 1) were performed with EZR version 1.52 (Saitama Medical Center, Jichi Medical University, Saitama, Japan), which is a graphical user interface for R version 4.02 (The R Foundation for Statistical Computing, Vienna, Austria). More precisely, it is a modified version of R commander designed to add statistical functions frequently used in biostatistics^31^.

### Measurement of biomarkers

All serum samples were kept frozen at − 80 °C until use. Assays were performed according to manufacturer’s instructions using the Bio-Plex Pro human chemokine panel (40-plex, Bio-Rad Laboratories, Hercules, CA), and the Bio-Plex Pro human inflammation 1 panel (37-plex, Bio-Rad Laboratories). Both assay kits include heterophilic antibody blocking reagents to inhibit the rheumatoid factor interference in the measurements. Bio-Plex Manager software (ver. 6.1, Bio-Rad Laboratories) was used to fit the calibration curve data using a five-parameter logistic regression model where recovery was in the 70–130% range, and to determine the observed concentrations of analytes in serum samples. For measured values out of range, the values above the upper and lower limits of quantification were replaced by the highest or lowest value available, respectively.

### Data normalization

For the sparse supervised projections to latent structures-discriminant analysis (sPLS-DA), the concentration data were normalised by log_2_ transformation and autoscaling (standardization). For classical receiver-operating-characteristic (ROC) curve analysis and multivariate exploratory ROC analysis and ROC curve-based model evaluation, the data were normalized by only log_2_ transformation.

### Multivariate data analysis

MetaboAnalyst 4.0 (http://www.metaboanalyst.ca/MetaboAnalyst/) and GraphPad Prism 8 (GraphPad Software) were used for statistical analysis of the Bio-Plex assay data. For volcano plot, the fold change using quantified concentration values of each analyte and *p*-value using standardization values between the relapsed and sustained remission groups were calculated using GraphPad Prism 9. *p*-values were adjusted by false discovery rate estimation using the two-stage linear step-up procedure of Benjamini, Krieger, and Yekutieli^32^ to determine the adjusted *p*-value. Each biomarker was analyzed individually, without assuming a consistent SD. Classical ROC curve analysis and violin plot were performed using GraphPad Prism 8 and area under the curve (AUC) was calculated separately for each biomarker.

### Protein–protein interaction network analysis

Cytokines with significant difference between the groups were used to elucidate protein–protein interaction (PPI) network, and KEGG analyses by STRING version 11 database (http://string-db.org)^33^. The PPI network was visualised using the Cytoscape software version 3.8.2 (http://www.cytoscape.org)^34^. The STRING functional enrichment outputs on KEGG pathway were visualised using GraphPad Prism 9.

## Supplementary Information

Supplementary Tables.
