# Supplementary material for: A novel multi-biomarker combination predicting relapse from long-term remission after discontinuation of biological drugs in rheumatoid arthritis
Source: Sci Rep. 2021 Oct 21;11:20771. doi: 10.1038/s41598-021-00357-9 (PMC8531387; doi:10.1038/s41598-021-00357-9)
Supplement: Supplementary file 1 — Supplementary Tables. [file 41598_2021_357_MOESM1_ESM.docx]

**Supplementary tables**

**Table S1. Measured inflammation and chemokine biomarkers.**

| **Bio-Plex Pro human**  **Inflammation 1 panel** | | **Bio-Plex Pro human**  **chemokine panel** | |
| --- | --- | --- | --- |
| APRIL/TNFSF13 | IL-27 (p28) | 6Ckine/CCL21 | IL-16 |
| BAFF/TNFSF13B | IL-28A/IFN-λ2 | BCA-1/CXCL13 | IP-10/CXCL10 |
| sCD30/TNFRSF8 | IL-29/IFN-λ1 | CTACK/CCL27 | I-TAC/CXCL11 |
| sCD163 | IL-32 | ENA-78/CXCL5 | MCP-1/CCL2 |
| Chitinase-3-like 1 | IL-34 | Eotaxin/CCL11 | MCP-2/CCL8 |
| gp130/sIL-6Rβ | IL-35 | Eotaxin-2/CCL24 | MCP-3/CCL7 |
| IFN-α2 | LIGHT/TNFSF14 | Eotaxin-3/CCL26 | MCP-4/CCL13 |
| IFN-β | MMP-1 | Fractalkine/CX3CL1 | MDC/CCL22 |
| *IFN-γ | MMP-2 | GCP-2/CXCL6 | MIF |
| *IL-2 | MMP-3 | GM-CSF | MIG/CXCL9 |
| sIL-6Rα | Osteocalcin | Gro-α/CXCL1 | MIP-1α/CCL3 |
| *IL-8 | Osteopontin | Gro-β/CXCL2 | MIP-1δ/CCL15 |
| *IL-10 | Pentraxin-3 | I-309/CCL1 | MIP-3α/CCL20 |
| IL-11 | sTNF-R1 | *IFN-ϒ | MIP-3β/CCL19 |
| IL-12 (p40) | sTNF-R2 | IL-1β | MPIF-1/CCL23 |
| IL-12 (p70) | TSLP | *IL-2 | SCYB16/CXCL16 |
| IL-19 | TWEAK/TNFSF12 | IL-4 | SDF-1α+β/CXCL12 |
| IL-20 |  | IL-6 | TARC/CCL17 |
| IL-22 |  | *IL-8/CXCL8 | TECK/CCL25 |
| IL-26 |  | *IL-10 | TNF-α |

* IFN-γ, IL-2, IL-8, and IL-10 are measured in both panels.

**Table S2. Sampling point and sample number.**

| **Sustained remission group** | | **Relapse group** | |
| --- | --- | --- | --- |
| **sampling point** | **sample number** | **sampling point** | **sample number** |
| A (study enrollment) | 14 | B (study enrollment) | 25 |
| G | 81 | E | 56 |
|  |  | C (just before relapse) | 23 |
| F | 14 | D (relapse) | 24 |
| total number | 109 |  | 128 |

Points A and B: study initiation; G and E: during remission; C: just before relapse; and F and D: study end for the remission group and relapse group, respectively

**Table S3. Summary of each feature in logistic regression model.**

|  | **Estimate** | **Std. Error** | **z value** | **Pr(>\|z\|)** |
| --- | --- | --- | --- | --- |
| (Intercept) | -21.228 | 8.818 | -2.41 | 0.016 |
| IL-34 | 0.791 | 0.255 | 3.11 | 0.002 |
| CCL1 | 4.252 | 1.832 | 2.32 | 0.02 |
| IL-1β | 0.465 | 0.264 | 1.76 | 0.078 |
| IL-2 | -2.42 | 0.887 | -2.73 | 0.006 |
| IL-19 | -0.180 | 0.182 | -0.986 | 0.324 |

The summary shows the contribution and significance of selected features in the multivariate logistic regression model using log-transformed analyte concentrations. The intercept is the value of the logit, or log (p/(1-p)), where p is the probability of becoming a relapsed patient, when the value of all the other terms in the model is 0. Estimate, estimated coefficient. A negative coefficient would indicate that the probability of becoming a relapsed patient decreases when the concentration of the marker increases. Std. Error, the standard error of the specified variable; z value or Wald statistic, which is exactly equal to the estimated coefficient divided by its standard error; Pr(>|z|), p-value given for the Wald test.
